# Photographic Illustrations of Skin Diseases

**Published:** 1888-07

**Authors:** 


Photographic Illustrations of Skin Diseases. By Geo. Henry Fox, A. M., M. D. Second Series. New York: E. B. Treat.

This work does not now need review, as it has been before the profession for several years. The second series is even ahead of the first in excellence; a great many new plates have been added and those which were imperfect have been replaced by better ones. The plates are simply superb; they are reproduced from photographs of selected cases, and colored by the brush of a skillful artist. As a consequence, they are of great value in teaching us the actual appearance of various skin lesions.

The text has been so greatly added to that it now forms a complete treatise on diseases of the skin.

Dr. Fox has achieved wonderful success as a teacher and lecturer, and this work contains his best thoughts.

